# ELDER MISTREATMENT SUBTYPES AND ANXIETY: DO DEFINITIONS MATTER?

**DOI:** 10.1093/geroni/igz038.2796

**Published:** 2019-11-08

**Authors:** Jieyang Zheng, Stephanie Bergren, XinQi Dong

**Affiliations:** Rutgers University, New brunswick, New Jersey, United States

## Abstract

Elder mistreatment (EM) and the magnitude of its relationship to anxiety may vary depending on definitional criteria. We leveraged data from the PINE Study, a study of 3,157 Chinese older adults in Chicago. EM was measured by 56 items on psychological, physical and sexual mistreatment, caregiver neglect and financial exploitation subtypes. Least restrictive, moderately restrictive, and most restrictive definitions of EM were constructed. Symptoms of anxiety were measured by the Hospital Anxiety and Depression Scale. Least restrictive (OR, 1.94; 95%CI, 1.57-2.40), moderately restrictive (OR, 1.56; 95%CI, 1.22-1.99), and most restrictive (OR, 1.39; 95%CI, 1.07-1.79) definitions of EM were all significantly associated with the likelihood of experiencing any anxiety symptoms. The magnitude of associations between EM and anxiety symptoms vary based on strictness of the EM definition. Future research should explore the potential causal relationships between EM and anxiety through longitudinal data.

